# Researches in Embryology, in Three Series

**Published:** 1842-10

**Authors:** 


### REVIEWS.

---

Art. III.—*Researches in Embryology, in Three Series.* By Martin Barry, M. D., F. R. S. E., Fellow of the Royal College of Physicians in Edinburgh.

These researches, though *three* in *series*, are *one* in *character* and design, constitute but a single Memoir, and are the product, almost entirely, of the scalpel and the microscope. The immediate subject of them, though not altogether new, is rare and curious. No American writer has ever seriously handled it—at least not extensively; nor, as far as we are informed, has any British one, except Dr. Barry, and the late Mr. Cruikshank.

To the physicians of continental Europe alone, especially to those of Germany and Prussia, and more recently to a few French physiologists, does the theme appear to be in any measure familiar. And even they, until the present century was somewhat advanced, had, in no small degree, neglected it, for considerably more than a hundred years—ever indeed since the time of De Graaf. But the study of it is not only revived; it is becoming active and even fashionable. Nor will the physiologists of England be indifferent to the example. For, throughout the civilized world, fashion controls science almost as much as dress. And, as an embryologist, Martin Barry, though comparatively but a young man, is already conspicuous.

Having qualified himself for the task, through reading and travel, by the acquisition of a thorough and correct acquaintance with all that had been previously written and known, respecting embryology, that ardent and enterprising student of nature entered on the further investigation of it, by analyses and observations of his own, with a degree of industry and zeal, energy and perseverance, that have rarely been equalled, and never perhaps surpassed. And the memoir he has produced sufficiently corresponds with the time expended and the skill and dexterity bestowed on it. For its length, it is one of the most elaborate essays in the English tongue—or indeed in any tongue with which we are familiar. And if it cannot be called a learned production, the reason is plain, and much more creditable to the author, than any amount of *borrowed knowledge* could make it, though dignified by the authoritative title of *erudition.* It occupies some ground previously unexplored, and therefore new, undescribed, and unrecorded. Yet it shows that, as relates to the subject of it, Dr. Barry's literary researches have been as extensive as they could be. For, as already intimated, that indefatigable inquirer has left, as we believe, no work unexamined, in any language, that could be made available in preparing him for his enterprise, or that could aid him in the prosecution of it. He has, therefore exhibited, in his investigations of this curious branch of science, all the learning attained or attainable, either by himself or any other person; because he has exhibited all that is extant.

But however interesting and valuable these "Researches" may be to the reader who attentively studies and thoroughly masters them, they constitute but an indifferent subject for a review. The reason is plain. They are already so dense a mass of facts, that it is scarcely possible to render them more dense. They cannot, we mean, by being thrown into the form of a summary, be reduced to so narrow a compass, as to be presented in substance to the reader at a single view. And were we to attempt to give him a competent acquaintance with the memoir, and thus do justice to its author by extracts from it, our review would be lengthened far beyond the usual extent of the articles admitted into this journal. The subject, moreover, is so intricate, and, to be fairly mastered and understood, requires such closeness and fixity of attention, that, curious and valuable in physiology as it is, we doubt whether it would be prized by a majority of our readers in proportion to its merit.

All we shall attempt therefore at present will be, to submit to the reader a succinct representation of the object aimed at, by Dr. Barry, and of the course pursued by him, in his attempt to accomplish it. This being done, we shall offer a few remarks on a point or two of doctrine embraced in his memoir, but not, in our opinion, correctly expounded. In this discussion we may probably offer some thoughts of our own on a topic connected with the subject of his researches, but not introduced into them. We allude to the process of menstruation.

The scheme of our author in his "Researches" is of a nature not easily defined, or characterised by words. Though strictly *elementary,* it is neither actually original, nor marked by any real profoundness of thought. Yet, pointing as it does to the very incipiency of things, it includes in it a spirit of enterprise so bold and aspiring, as to be hardly unworthy of the epithet sublime. This is the more especially true, from the consideration that its aim is to throw light on a subject which was once deemed inscrutable. And the difficulty of the project may be almost said to have justified, at the time, an entire disbelief in its practicability. But time and means, judgment, resolution and patient labor are all but omnipotent, in the affairs of man. There are few *earthly* ends that they will not attain, if, under such regulation and direction, perseverance be pushed to the requisite extent. Of this truth Martin Barry stands forth himself an eminent example.

The Doctor was not merely content to *begin his labors,* where preceding inquirers into embryology had *closed theirs.* That step, so decidedly unambitious, would have deprived him entirely of all bold and lofty pretension to originality, which he was unwilling to resign, and would have rendered him, by his own acknowledgment, a subordinate writer. He therefore ascended, or at least endeavored to ascend, as his point of departure, to the very fountain-head of organic existence.

Commencing with the real seminal or primitive animal matter, even before it had become organized, he has traced, with uncommon minuteness and care, and also with great apparent accuracy, its progress through all its subsequent changes of size and figure, and in all its multiplied and diversified elements, until the complete formation of the ovum, its entrance into and progress through the fallopian tubes, and its lodgment in the uterus. And there is good reason to believe, that, in his close and keen examination of these mutations and movements, he has not only made several interesting discoveries of his own, but also corrected certain errors contained in the writings of preceding inquirers, and thus doubly contributed to the advancement of embryology.

His observations, it is to be understood, were made almost exclusively on the mammiferous class of animals. And he believes (we doubt not correctly) that he has settled the question (if indeed it were not previously settled) as respects the place both of the *formation* and the *impregnation*, of the ovum, in that class. The ovary he considers the site of both processes. And that those processes occur in the same place in all viviparous, and perhaps in all oviparous animals also, is hardly to be questioned.

In alleging that Dr. Barry has "*settled*" the question respecting the site of the formation and impregnation of the ovum, we do not claim for him any thing new. We attribute to him only the full and satisfactory confirmation of the opin ion of another. By those who have paid attention to the subject it is known, that upwards of a century and a half ago, De Graaf pronounced the ovary to be the seat of both these processes. The opinion, however, was subsequently opposed by Haller and his followers, who contended that the fallopian tubes were the places where the ovum was formed out of matter derived from the ovaries, and deposited in the tubes in a shapeless and immature condition; and that impregnation was effected in the same organs. In this opinion we say Dr. Barry not only concurs with De Graaf, but has established its truth.

For the benefit of those who may wish to repeat his experiments, it is necessary to mention, that our author conducted his researches chiefly by the dissection and inspection of female rabbits, during the period of their rut or sexual desire, especially just before and after the act of copulation. And he was so fortunate as to collect evidence still farther confirmatory of the fact, well known before, that the rutting or amative season of the mammaliæ, is uniformly accompanied by an augmented vascularity of the ovaries, and other parts of the generative system, as well as by a preternatural accumulation of arterial blood in them. This phenomenon in the females of the inferior animals, as will hereafter be made more fully to appear, is analogous to menstruation in woman, and is no doubt designed to subserve the same purpose—the preparation of the generative organs for the process of impregnation, and, in woman, for the continuance of them in that condition.

As a farther element of the history of the memoir we are examining, it may be mentioned that it first appeared as a communication to the Royal Society of London, and was printed originally, and we believe exclusively, in the Transactions of that distinguished body. The plates contained in it are very numerous, of great diversity, and highly finished. They are also of great value, as without the demonstrations they afford, the text of the work would be unintelligible. Such, as we are told, was the estimation set on the memoir, by those appointed to judge of its merit, that it opened to its author the door of the Royal Society, of which he is now a member. So much for all the general remarks we deem it requisite to make on the memoir.

We have alluded to a point of doctrine maintained by our author, in the soundness of which we do not concur with him, and which we have promised to make the theme of a brief discussion. It is contained in the following extract from the memoir.

"As to the particular *locality* in which the ovum becomes susceptible of development [*is impregnated*] physiologists are not agreed. Some maintain that it acquires this susceptibility before it leaves the ovary; others that the change is not effected until after its expulsion from that organ.

"It is not my intention te discuss the question whether contact of the seminal fluid with the ovum is or is not essential to impregnation. Yet perhaps it may be proper to refer to the *possibility of that contact*, while the ovum is still in the ovary, that having been denied."

To demonstrate this possibility, and we may say, to show his *belief* in the *actual occurrence* of the contact which he pronounces *possible* (for such belief he virtually avows) our author continues.

"In *seventeen* out of *nineteen* instances of the rabbit, though the parts were generally examined while warm [*immediately* after the death of the animal—and also soon after coition] I was unable to discover spermatozoa in the fluid collected from the surface of the ovary. In the other *two* instances, however, spermatozoa, or at least animalcules exactly like those I had been accustomed to meet in the uterus and vagina, were really found in the ovary."

That the ovum is impregnated as well as formed in the ovary, is a point of doctrine, in the truth of which we have long believed, and have often defended it, for the most part *orally*, but also through the press. The striking changes which take place soon after coition, in one or more of the ovarian vesicles, previously to any discharge from them, furnish abundant evidence of the fact. But that the semen masculinum, whose influence is essential to impregnation, makes its way to the ovary, and, in its formal and entire character, comes into immediate contact with the ovum, or containing vesicle, we deem not only doubtful, but altogether incredible. And though we do not pronounce the event *impossible*, we regard its improbability as of the highest order—of the same order indeed with that of the spontaneous *ascent* through the air of a mass of iron, or the *descent* of a volume of hydrogen gas without any compulsive force, the atmosphere being tranquil, and in a natural condition.

The cause of our disbelief, moreover, seems to us reasonable and plain. We consider every positive and well-established fact of the case as testifying strongly against the hypothesis. Nor, of course, if this be true, is there, or can there be a law of animal action in favor of it.

Some of the facts alluded to shall be presently cited. Previously however to this, we shall specify the principal modes, by which the semen masculinum is supposed to be brought into contact with the ovarium, and state our objections to them.

1. Some physiologists (perhaps a majority of those of the present day) contend that that fluid is introduced into the uterus, during coition, *vi jaculationis penis masculini;* and that thence, by some *secret* spring of movement (for none such has yet been discovered, nor scarcely even fancied) it glides or hurries forward, enters and passes along the fallopian tubes, issues out of them again, and finally reaches the surface of the ovarium. Nor is its journey yet terminated. It penetrates the ovum, and comes into immediate contact with what our author terms the "germinal vesicle;" and there the work of impregnation is completed.

Such is the darksome journey—half by compulsion—half spontaneous—supposed to be performed by the semen masculinum, in quest of the place of action which awaits it. And that it is an arduous one, the most sanguine of its advocates must acknowledge. To us the series of obstacles opposed to it, even when the generative organs are in a sound and perfect condition, appears to be insuperable. And under the influence of certain derangements of them, which not unfrequently occur, without preventing the process of impregnation, the impediments to the passage of the fluid toward the ovarium are so much augmented, that, in her efforts either to remove or surmount them, even fancy herself, from a lack of expedients, must falter and fail. Of the difficulties which thus present themselves, when the generative organs are in a healthy condition, the following are a few.

The axes of the vagina and the uterus do not so correspond in direction, as to meet and constitute a straight line. At their junction with each other they form an angle, which, though far from being acute, effectually prevents the semen masculinum from being projected into the *os tincæ,* and through the neck and strait, into the cavity of the uterus. The semen, when ejected in coition, may strike the uterine cervical extremity; but that it cannot enter into and pass through it, every fact bearing on the subject conclusively shows.

In the virgin state of the female generative organs, the rugæ formed by the lining of the vagina constitute another obstacle to the projection of the semen. Nor is this all. The viscosity and weight of that fluid, united to the adhesiveness of the mucus spread over the lining of the whole canal forms a further very serious obstacle to its projectility through a crooked and very narrow opening, or rather through no actual opening at all (for the sides of the canal are in contact) into the cavity of the uterus. In truth it appears to us, that a more impracticable and hopeless project can hardly be imagined than that of introducing the seminal fluid, during coition, *vi jaculationis,* into the uterus.

But admit the difficulties of the introduction to be vanquished, very little is yet done toward the completion of the enterprise. At least the most arduous portion of it remains to be performed. The fluid has lost the whole impetus or force of movement received during coition from the male organ. Nor does there exist, in the surrounding parts, a single organ or portion of machinery, to communicate to it a fresh impetus. It must first, by some instinct and locomotive power of its own, grope its way to the fundus uteri, there search out the scarcely more than microscopic orifices of the fallopian tubes, enter them, and then crawl along those lightless canals, until it shall finally arrive at the ovarium, there to aid in the perpetuation of our race. We say *our;* for our observations relate to human impregnation. Yet are they applicable to that function on a much broader scale—probably to *universality itself*.

But, according to the tenets of the hypothesis we are examining, the travels of the semen masculinum are not at an end, even when it has perched on the ovarian vesicle which contains the ovum. It must pierce through the parietal membrane of the vesicle, as well as through the proper membrane of the ovum, before it can operate effectively on the "germinal vesicle" of that body. And, if we mistake not the hypothesis, on that it must operate, else impregnation does not follow. *The contact must be complete.*

That the discussion may be conducted, on our part, with the utmost fairness and liberality, we again admit what we consider inadmissible, because we conscientiously believe it can never occur. We concede that the seminal fluid has not only reached the fundus uteri, but that it has made its way into the minute orifices ef the fallopian tubes. But how is it now to continue its journey, and finally reach its place of destination? The tubes can by no possibility conduct it there. They are in the condition of a broken-down bridge, which positively refuses to the traveller a passage. To drop figurative language, and express ourselves in that of our profession, the fallopian tubes are in a *flaccid condition*. But when in that state, they do not, by a considerable distance, extend to the ovaries, and cannot therefore convey to them and deposit on them aught they may contain. To perform that office they must be in a state of *vital erection*, prepared for efficient functional action. For surely our opponents will not contend that the seminal fluid, by being merely admitted into the fallopian tubes, passes through them without their aid, and equally without their aid, makes its way to the ovary. Yet notions no less preposterous have had their advocates. Nor are we by any means confident that fancies as visionary, respecting impregnation, are not now entertained and defended by individuals who rank with the gravest and wisest of our philosophers.

Does any one ask us how we know that the fallopian tubes are flaccid during sexual intercourse? To this our reply is prompt and positive. We know it because it has been ascertained by observation. And Dr. Haighton, one of the first physicians of his day, was the experimenter and observer. He killed a female rabbit, when in full rut, *immediately after coition*, and the fallopian tubes were completely flaccid. Nor did they exhibit the least sign of having been recently rigid, or injected with blood. He then proceeded to kill other female rabbits, also in rut, at every hour after coition, until the *ninth* hour, when incontestable signs of impregnation already presented themselves; and the fallopian tubes were but just beginning to prepare themselves to pass into a state of rigidity. They were beginning, we mean, to be preternaturally injected with arterial blood. And wherefore were they so? The cause may be easily rendered. Their action was to be soon required to receive, convey to the uterus, and there deposit, the contents ef some ripe ovarian vesicles, which were preparing to burst, and commit to the fimbriated extremities of the tubes the matter within them. These experiments were several times repeated with the same results. Nor have either these, or the many other experiments of Dr. Haighton respecting impregnation, though cavilled at and inveighed against, ever been subverted or in the slightest degree invalidated by the experiments of any other writer on the subject. Indeed they cannot be subverted, for the best of reasons. They have been conceived with judgment—and accurately performed; and their foundation is truth.

To speak of this matter plainly and irrespectively, and apply to things the terms that truly expound and graphically characterize them. The circumstances of the case compel us to regard as essentially preposterous that hypothesis which contends that the semen masculinum passes into *positive contact* with the ovarium. Once more we emphatically ask, and solicit to the question the serious and deliberate attention of physiologists—by what means is the passage effected? or, with the present organization and endowment of the parts concerned, by what means can it be effected? And we challenge a calm and philosophical reply—a reply abounding in fact, reason and argument, and free from conjecture, assertion, and fancy.

That by the projectile power of the male organ, in the act of coition, the seminal fluid can be thrown into and through the uterus and fallopian tubes, and thus be brought into actual contact with the ovarium, we again declare to be *physically impossible.* Admit, we repeat, that it can be thrown into the uterus, *vi jaculationis;* it can be thrown no further. As far as the original impetus is concerned, there it must stop. How then is it to be put in motion again, and conveyed to the ovarium, its supposed place of destination? The uterus most assuredly has no apparent fitness, in either structure or habit (as far as we are apprized of its habits), to conduct it thither, in a course of motion directly the reverse of that which its customary action produces. For it need hardly be remarked by us, that the usual, we believe the *universal* direction of the action of the uterus is from the fundus to the neck—not from the neck to the fundus. But did it convey the seminal fluid to the openings of the fallopian tubes, the latter must be the course of action taken on by it; because no other would produce the desired effect. Nor were the fluid actually introduced into the uterine end of those tubes, do they appear to be in the slightest degree qualified to convey it to the ovarium. Why? Because the action suited to such conveyance would, in its direction, be exactly the reverse of that which they are positively known to perform. Matter has been often found in them, in its passage from their fimbriated extremities to the uterus, but never journeying in the opposite direction. Never has any substance been detected in them *certainly* passing from their uterine to their ovarian ends. All allegations to that effect are but unsubstantial guess-work.

It is definitively known, we say, that the fallopian tubes carry matter from the ovarium to the uterus. Were it equally certain that, *when in a healthy condition,* those same tubes carry matter, by their *natural* action, in the opposite direction, the fact would constitute a phenomenon *unique,* as we believe, in human physiology. We remember, at present, no other instance, in the system of man, where a tube, by its natural and healthy action, is alleged to convey matter, either solid or fluid, in opposite directions, at different times. The healthy functions of our systems are coincident and harmonious. If, in any case, contrariety or clashing occur in the economy of our bodies, it is the result of a condition of things neither sound nor natural. The mere fact that the cystic bile *twice* traverses the cystic duct—first toward the gall-bladder, and then toward the duodenum, can with no force or justice be cited in contradiction of the general truth in physiology here contended for.

Will any one allege that the spermatic animalcules—the *homunculi* of former writers, and the *spermatozoa* of those of the present day—will any one fancy or even dream that those microscopic specks of matter, containing within themselves the impregnating and quickening leaven of vitality and being, and possessed of muscular or some other sort of locomotive powers, crawl, and grope their way, from the vagina, or the neck of the uterus, through the worse than Cretan labyrinth of the generative apparatus, until, without either chart, compass, or clew, they arrive at the ovarium, enter into the ova, complete the work of impregnation, and, becoming themselves embryos first, and infants next, are ultimately developed and matured into adults? Will any one peril his reputation for intellect, by avowing himself the advocate of a notion like this? We think not. Yet would the fancy, wild and visionary as it is, be just as consistent with reason and common sense, as any of the elements of what may be called significantly enough, the CONTACT HYPOTHESIS—of that hypothesis we mean which, in its scheme of expounding things, attributes to *contact* an influence and a consequence far beyond what it possesses—which, in particular, doggedly maintains, that of bodies in contact the mutual action is not only much more certain, but also much more *explicable*, than of those that are not in contact—and which, in fact, virtually contends, that their mere *being in contact* is of itself a satisfactory reason *why* bodies

*do* act on each other, and their simply *being apart,* a sufficient season why they *do not.* But of this *contact-hypothesis* due notice will be taken hereafter; while, in the mean time, we shall proceed in the discussion of our more immediate subject.

The foregoing are some of the difficulties to be encountered by those who would conduct the semen masculinum to the ovarium, the genital organs of each sex being in a natural and healthy condition—such a condition, we mean, as offers no impediment to their most efficient action. And the difficulties of the case are greatly augmented, in magnitude at least, if not also in number, by various derangements which occur at times in the organs, while the power of propagation remains, *certainly undestroyed,* if not unimpeded. Of such derangements the following may be noticed.

The *projection* of the seminal fluid into the uterus is augmented in difficulty by the *truncation* or shortening of the penis, by the loss of a large portion of its entire body, as well as by strictures of the urethra, which scarcely allow even the urine to pass. And such *projection* is rendered impossible by anomalous openings of the urethra, not at the point of the glans, but on the underside of the penis, *behind the glans.* Yet, while laboring under these embarrassments, men have often had children born to them. Instances of the kind have fallen under our own notice, while, as related to the fidelity of the mothers, no shade of suspicion was entertained.

But the most hopeless obstacle to the passage of the semen masculinum to the ovarium remains to be mentioned. It is the entire occlusion of the vagina, the os tincæ, or the neck of the uterus. This impediment, absolute while it continnes, is effected in several ways; by imperforated hymeneal membranes, which have unquestionably existed; by morbid adhesions, the product of inflammation, which have also existed; and by tumors arising from various causes.

That under the obstruction to the passage of the seminal fluid into the uterus, offered by these anomalies, impregnation has been effected, we are forbidden to doubt. Testimony to that effect is transmitted to us, on authority as high and valid, as any which the annals of our profession afford. Among the individuals who have recorded themselves as witnesses of such occurrences may be mentioned Harvey, Morgagni, Hildanus, Ruysch, Simpson, and Haller—men whose veracity has never been impeached; whose competency to investigate and judge has never been questioned; and whose contributions to medicine have immortalized their names. Nor is this all. Several strongly attested cases, of a more recent date, are publicly recorded, and have been reported to *ourselves* in private letters from individuals both of intellectual distinction and high moral standing; and we have in person witnessed a case, on which we could put no other construction. But to close our descant on these difficulties, all of which are formidable, and some of them insuperable, and pass to another subject.

Perhaps there is no exercise of the mind in which a greater display of *false philosophy*, or as it might well be denominated *philosophical folly* is made, than in that of stubborn adhesion to what we have called the CONTACT HYPOTHESIS. And more or less abundantly that hypothesis throws its influence into every profession, and every department of human research.

The medical adherents to it, who constitute a large majority of the Faculty of christendom, can neither comprehend, nor apparently believe in the action of medicinal substances, except they be in some way, in their mode of employment, connected with *contact*. Hence, if such substances prove in any degree effective, they are believed to be dissolved or mixed in the blood, which conveys them directly to the deranged portions of the body, and thus enables them, through immediate contact, to act on and cure or relieve their disease.

For this high efficacy and influence of contact these gentlemen contend, without having, in many cases, the slightest evidence that such contact exists (for the medicinal substances which they believe to be mingled with the blood cannot be detected there), and without forming a *definitive*, or, even tolerably *exact* idea of the import of the term.

Strictly speaking we are hardly if at all authorized, *as philosophers*, to say that any bodies, of which we have definite knowledge *are in mutual and positive contact*. We can pronounce them only *near to each other*. Why? Because bodies of any appreciable size can, by certain powers and processes, be brought still nearer to each other, than they are in what is called a natural state. And the same is true of even the minute constitutional particles of matter existing in masses. They can be still more closely approximated to one another. In other words, each mass can itself be more consolidated, which implies an increased proximity of its particles. As relates to bodies not brittle, more especially if they be possessed of life, this is certainly the case. Their constituent molecules can be brought nearer to each other than they are in their usual condition. Of all masses of fluid matter, quicksilver itself not excepted, the same may be affirmed. By compression and otherwise the nearness of their elementary atoms to one another may be increased. A few further remarks on the term *contact* may aid us therefore in rendering our views, in the present instance, the better understood.

In preciseness of expression, contact is undefinable; because it has no definite and positive meaning. It is an abstract or general term bearing relation to space, and indicating degrees of proximity that may be pronounced almost innumerable, and therefore exceedingly different from each other under different circumstances, and in different cases. It might be called, with sufficient significancy, the PROTEUS of the English language. Were we to attempt to affix to it any settled definition, after having pronounced it "undefinable", we would say that it signifies (if indeed it can be made to signify any thing) the shortest space that does or can exist between two bodies—or the shortest space we can measure in fancy. Like the notion of the infinite divisibility of matter, it is an abstract truth, but a practical fallacy. On this abstruse and interesting point we shall only add, at present, that notwithstanding all that is said about contact and its effects, it is almost certain, that between solid and ponderable bodies, it *has no existence*. Such bodies appear to be kept apart by a subtle, elastic and imponderable aura; which extends to all places, and penetrates all things, but of which nothing is known except from its effects. Might we coin a word suitable, as we think, to the present occasion, we would say, that, as relates to both massses and molecules of *ponderable* matter, this *imponderable aura* seems to act the part of a universal *betweenity*—a substance which lies between them, and so far separates them from each other, as to allow them room for some *sort of action*—for we cannot doubt that, more or less, *every atom of matter acts a part* in the scheme of creation. In a system marked by positive and *consummate wisdom and perfection* (and such is and *must* be the handiwork of an All-perfect being), there can be no *idleness.* For idleness is essentially *imperfection.*

If all this be true then (and the refutation of it is fearlessly challenged), how fallacious are the views, and how futile the labors of our *contact*-philosophers, who conceive themselves in possession of a talisman, competent to the solution of some of the most obstinate difficulties in physical science! And that talisman is *contact.* And that contact is nothing more than an *imaginary or supposititious* relation. Yet do those mistaken inquirers seem really to believe, that if they can bring two bodies together, and place them in what they call *contact,* they fully understand and can clearly expound the reason and mode of their operating on each other; and that if the bodies be *not* thus brought together, they cannot operate on each other at all.

Such we say appears to be the groundless notion of those votaries of contact, while it would be easy to show, had we leisure to dwell on the subject, that it is by the mutual influence of bodies *not in contact,* but remote from each other, that the economy of nature is chiefly carried on. In proof of this we might refer to the organization and movements of the heavenly bodies, which are maintained and regulated exclusively by *remote agency.* The agents in this magnificent and sublime department of creation, are termed gravitation and repulsion, or rather the principles of those high and important forms of action. And to the natural and effective operation of those principles, distance between the bodies acting on each other is essential. Were the several orbs which belong to the solar system, and those still more remote from us in space brought into contact or close proximity to each other, there is reason to believe that the economy of creation would be disastrously changed; and that little short of general ruin would ensue, and a new order of things succeed to the wreck.

When we advert to terrestrial matters, and take a survey of them, phenomena similar in character, and governed by similar principles of action, every where present themselves to us. In confirmation of this we may again refer to gravitation and repulsion, in all their multifarious effects and bearings, and also to the workings and manifestations of magnetism, electricity and galvanism. In the production and play of the phenomena proceeding from these agents, bodies remote from each other are in all instances and necessarily concerned; and in none of them is contact either indispensable or useful. On the contrary, it would derange and confound the whole scheme of action, now so beautiful, harmonious, and perfect. And there is reason to believe, that the same all-pervading, subtle, and ever-active aura, to which reference has been already made, is essentially influential in the control of the pageant.

But we have not yet closed our remarks on the influence and operation of distant bodies on one another. Far from it. The most striking and appropriate to our present purpose are yet to come. And they will bear relation to living matter, especially to the human system, in the performance of its functions in both health and disease. The following are a few of the facts by which our position is sustained.

When wine or other cordial and exciting articles of food or drink are received into the stomach of persons exhausted by long fasting, fatigue, or otherwise, they throw their influence *instantaneously* throughout the entire systems of those who swallow them, and refresh and invigorate the most remote in common with the most proximate organs of their bodies. And this they do long before it is possible for them to reach in snbstance, through the circulation of the blood, the portions of the system which they thus revive. Certain poisonous substances in like manner, if introduced into the stomach, or even applied to the lips, tongue, or eyes, act with equal suddennesss on the most distant parts of the body, and produce death. Nor is this all. Deleterious impressions made on the skin by cold, burning, or in any other way, derange by sympathy the internal or remote parts of the body as certainly, and almost as speedily and seriously, as if they were made on the sympathizing organs themselves. And irritation on the lower extremities is known to have produced gastric spasms, tetanus, mental derangement, and death. Innumerable other instances moreover of a like description might easily be adduced.

Actual contact then between bodies, whether it be positively producible or not, is not necessary to their acting on each other. As far as we are informed on the subject, this is true of the entire scheme of material creation. But it is pre-eminently so, as relates to the economy of man, and other forms of living matter, in which all action, whether natural or unnatural, healthy or morbid, is the product of *stimulation* —the product, we mean, of an influence wholly unknown to *dead matter*, and of which dead matter is unsusceptible—the product of an attribute peculiar to *living* matter, and which is as entire a stranger to chemistry, as chemistry is to mathematics or theology. And it is perfectly well known that for the production or change of action in a part, whatever may be its character, *remote stimulation* is as efficacious as *proximate*. Our meaning will be understood to be, that an organ may be, and often is, as fully and effectually excited and thrown into action, by suitable impression on a distant organ, as it can be by impression on a contiguous one, or even on itself.

On the authority of the foregoing facts, then (and many others we say of like import might be cited), we confidently repeat, that, to whatever extent appearances and even authorities may testify to the contrary, *contact* between bodies is not indispensable to their highest and most efficient degree of action on each other. In many cases it is unfavorable to such action, and in not a few entirely preventive of it. In a special manner it furnishes no facility in detecting the causes of action; or in giving any correct philosophical exposition of it. It enables us to assign no reason, why, on scientific grounds, the action may not have as certainly occurred without contact as with it.

It has long been matter of surprise to us, especially as relates to living beings, that this truth, obvious and incontestible as it is, should be so completely overlooked. For overlooked it is by almost every one—the cultivated and enlightened portion of the community forming no exception to the oversight, any more than the uncultivated and ignorant.

The universal impression is, that, provided an agent, whether salutary or deleterious, be brought into contact with any organ or part of the body, all difficulty, as respects causation is removed, and the reason why such agent produces a given effect on the part it touches completely understood. And *mere contact* is the talisman honestly though heedlessly believed to solve the difficulty, and render obvious to every one the entire cause of the action produced, and the result that follows. Yet to the philosophical inquirer and the clear and able thinker a moment's serious and strict examination cannot fail to render it certain, that the simple contact of bodies does no more toward the disclosure of the cause why they act on each other, nor toward the exposition of the mode of their action, than would their remoteness from one another do, or than would any other practicable posture, bearing, or condition of them do. For we repeat what we have heretofore stated in substance, that as regards a fitness to disclose the cause of the action of one body on another, *contact* and *remoteness* are in a like degree *nullities.* It is the *properties* of bodies, not their *positions* or mere *exterior relations* that fit or unfit them to act on one another. So much for our general doctrine. Now for its application to the subject of our inquiry.

Notwithstanding the foregoing truths, which, though we may *deem* them incontrovertible, we do not *dogmatically proclaim* them so, nor claim or even solicit for them aught save a deliberate and impartial examination—the stricter the better—notwithstanding these considerations, most physiologists seem to think, that if they can succeed in showing that the semen masculinum, positively finds its way to the ovarium and touches it, they have explained satisfactorily the process of impregnation. The mere coming together of the male and female products appears to be to them sufficient for the purpose. It would seem, in their estimation, to explain to them the mode of operation of the two substances on each other, and to reveal to them the causes of the events that succeed. So deep is the deception which these physiologists practise on themselves, as well as on the public, by the negligence, and superficialness of their mode of research! Those gentlemen moreover under the influence of a quixotic spirit, seem pleased with the pastime of creating or imagining difficulties, and placing them in their own way, that they may display their prowess and dexterity in attacking and vanquishing them. In the present case they have two difficulties—one of them *artificial*, being needlessly manufactured by themselves, and the other erected by the hand of nature. The first is the difficulty of conducting the semen masculinum to the ovarium; and the second, the difficulty of knowing how to dispose of it on its arrival, and to understand and expound the office it has to perform. Nor would the latter difficulty be at all diminished could they actually bring the seminal fluid to the ovarium, and diffuse it over the surface of that organ, or even introduce it into its interior. They could as easily discover and explain its mode of operating and producing impregnation, when at a distance, as when thus in contact with the ovarium, or even when within it. Their hypothesis is in no way or degree benefitted therefore, by the more than herculean task of conveying the seminal fluid to the female organ, where they are ambitious to place it.

We, on the contrary, content with encountering a single *natural* difficulty, create unnecessarily no *artificial* ones.

Hence we decline the needless, useless, and impracticable labor of conducting the semen masculinum to the ovarium. We have therefore to contend with only the simple natural difficulty of expounding the influence and operation, *by sympathy*, of the semen masculinum on the ovary and its contents. And that we make no effort to expound, convinced of the impracticability of the task. We only admit the process as a fact, and illustrate and sustain, as best we may, our views in relation to it, by means of analogy. We repeat however that there is no more difficulty in accounting for the sympathetic influence of the semen masculinum on the ovarium, when deposited at a distance from it, than there would be, were it applied immediately to its surface—or even introduced into an ovum and brought into contact with the "germinal vesicle." And to make good their enterprise, we say again, the *contact-defenders* must thus introduce it, else their labor is lost, and their scheme defeated. Of the sympathetic theory of impregnation more will be said hereafter in detail.

Another class of physiologists equally bent on bringing into contact, in the work of impregnation, something of the masculine, and something of the feminine gender, have recourse to their fancy, and, by the creativeness of that, produce what they denominate a seminal aura. This they derive from the semen masculinum deposited in the vagina, and conduct it through some route (no matter what) to the ovarium, where, by contact with the ovum, or rather by making its way into it, it effects impregnation. But this aura exists only in the notions of visionaries. Indeed the whole hypothesis is but a mere creation of fancy, and shall therefore be dwelt on no longer, as a subject of discussion.

But we are not yet done with this phantom-tale of *contact*. A third set of physiological schemers, resolved on sustaining their notion, and making it subservient to a favorite hypothesis, convey in imagination the semen masculinum to the ovarium by a third and more circuitous channel. They contend that that fluid, being absorbed from the vagina, where it is deposited, *during coition*, enters into the blood vessels, mingles with the blood, and, by that fluid, in the course of its circulation, is brought into immediate *contact* with every part of the body, and of course with the ovarium, where it effects impregnation.

To every one who may seriously reflect on it, the fallacy, not to say absurdity of this hypothesis, cannot fail, we think, to be obvious and glaring. Were it true, the case would be alarming to virtuous women; because the effect might be the peopling of the world with swarms of *illegitimates.* The entire mass of blood in the impregnated female would or at least *might* be imbued with the male seminal fluid, to such an extent, as to render it contagiously operative on other women as well as on its owner. In such a state of things, simply to introduce into the veins of an unimpregnated female a portion of the blood of a pregnant one, would produce in the former a condition similar to that of the latter. In plain terms it would impregnate her also. Thus might animal reproduction be promoted, like small-pox, or the vaccine disease, by simple inoculation. At least, ludicrous as the fancy may be thought, we perceive no good reason why the event might not occur, could the whole mass of a woman's blood be so saturated with semen masculinum, as to impregnate herself. But we must dwell no longer on so dreamy a conception. There exist many other hypotheses of impregnation, founded also on the notion of *contact*, all of which are untenable, and some of them, especially that of Buffon, wild even beyond the fictions of romance. In the citation and discussion of them therefore we must consume no more time.

Having thus avowed our dissatisfaction with all the *contact-hypotheses* on impregnation, were we asked, in what theory we are ourselves believers? we would reply; in that which *rests on the doctrine of sympathy.* But we shall speak of it cautiously. Our meaning therefore is, that we give a preference to that over all other doctrines, without positively averring it to be true, beyond contradiction, doubt, or cavil.

Of this theory the ablest and most laborious advocate and sustainer was Dr. Haighton. And to us his experiments on the subject are as satisfactory and conclusive, as are those made by any inquirer, or on any physiological theme, that can be named.

It is a matter of regret to us that a want of room forbids us to submit a detailed account of those experiments to the scrutiny and judgment of our readers, and compels us to rest content with laying before them, in general terms, a brief exposition of the principles of the theory. And that this exposi tion may be the clearer and more definite in its elements, and the better understood, a few preliminary remarks will be offered.

That no mistake may exist as to the exact meaning of sympathy, as to be here interpreted, it is requisite for us to observe, that it is a term employed to denote the fact, that an impression made on one part of the human body, when possessed of life, produces excitement and action in another part, near or distant, as circumstances may direct. And the kind of action excited depends chiefly on two contingencies—the nature and character of the exciting body—and the organization and nervous endowment of the part that acts. Something also depends on the condition of the body as relates to health. In a particular manner the production of a specific effect requires the influence of a specific cause.

A well known fact in physiology, interesting in itself, and important to be borne in mind in the present discussion is, that sympathy in woman is much more vivid and powerful than in man, and therefore capable of the production of a deeper result. And another fact equally well known is, that the *genital* system of woman controls more powerfully her *general* system. To these facts are to be added another of equal certainty—that each portion of the female genital system sympathizes strongly with every other portion of it; and that it is these cords of mutual sympathy which *constitute it a system.* These positions are to be regarded as so many fundamental truths in physiology, and as such to be remembered in the sequel of this discussion.

Another important physiological truth to be also borne in mind, on the present occasion, is that the ovarium is essentially a glandular body, capable, like all other glands, of producing out of the blood something which the blood itself does not contain in a formal state. By means of this *creato-constructive* power (might we compound a term expressive of our own opinion) the ovary gives being to the ovum, in all its elements, which, *substantively* but not *formally*, had been previously in the blood.

A farther truth of interest and moment to us is, that, as already stated, vital action is the result of *stimulation*, and without stimulation of some sort could never be produced. The notion that it is or can be the result of any form of chemical agency, is as utterly absurd as would be the ascription of mechanical products to *moral means.*

Of stimulation it may be correctly enough said that there are two classes or kinds—*proximate* and *remote.* Of these the former arises from impressions made immediately on the stimulated part, while the latter is the product, *through sympathy*, of an impression made on a distant part. And in various instances, each sort of stimulation excites *secretory* and *nutritive* or *formative* action in perhaps an equal degree. As a general rule, however, there is reason to believe that remote stimulation is most efficient—ministers, we mean, to the performance and steady maintenance of the greatest number of important functions. This truth, too, our readers will do us the favor to bear in mind.

To the production of the ovum, both secretory and formative action are essential. By the former the requisite materials are prepared from the blood. By the latter those materials are constructed into the ovum. We shall only add, in this place, that secretory and formative action are excited by remote stimulation, as certainly and effectively as by any other kind—very often much more so. In the following instances the truth of this position abundantly appears.

1. *Secretion of tears.* This secretion is excited by those impressions on the brain (for they are nothing but forms of cerebral excitement) called *joy* and *grief*. It is also excited by irritating impressions on the eye-balls and eye-lids—and in children by pain in any part of the body—in each case the lachrymal gland being at a distance from the stimulated point. Were the primary irritation made on the gland itself, there is no reason to believe that the result would be a secretion of tears.

2. *Secretion of saliva.* This is excited by impressions, whether agreeable or disagreeable, on the tongue, lips, and palate—as well as by the sight and odor of agreeable food, and the remembrance and conception of savory substances. Yet in neither case is the salivary gland proximately excited. The excitement it experiences is exclusively sympathetic.

3. *Perspiration or sweat.* This cutaneous discharge is excited by an impression on the stomach, produced by liquids either cold or hot. When a person heated by hot weather or severe exercise swallows hastily a draught of cold water, his skin secretes promptly an abundance of sweat. On the same principle of sympathy an immersion of the feet and ancles in warm water often excites perspiration over the whole body.

4. *Secretion of bile.* This secretion is excited through sympathy by an impression made on the mucous lining of the stomach and intestines, by calomel, aloes, and sundry other medicinal agents. Nor have we the slightest reason to believe, that an impression made immediately on the liver, by sprinkling the same articles over its surface, or injecting them into its blood vessels, would be equally effective in the production of bile. On the contrary, hepatitis, accompanied by a suppression of the biliary secretion, would much more probably be the result of such an experiment.

5. *Secretion of urine.* Of this secretion the same may be said. It is best promoted by remote stimulation. An impression made on the stomach by ripe water-melons produces frequently, and in a short time, a copious flow of liquid from the kidneys. The same effect is often induced by a draught of cold water alone—by water impregnated with some vegetable acid, or by the application of cold water to the surface of the body.

Of the secretion of semen masculinum we forbear to speak in detail, else could we easily show, that it also is essentially connected with remote stimulation. And of the pancreatic, the serous, and the mucous secretions, it would be easy to make it appear, that the same is true.

Exemplifications of formative or constructive action show themselves abundantly in the general *nutrition* of the body, and in all sorts of tumors, and other forms of unnatural growth.

The foregoing are the principal kinds of secretive and formative action in the body. And that they are all promoted, and may be greatly augmented, by remote stimulation, no physiologist will venture to deny. Nor can he refuse to acknowledge that to proximate or immediate stimulation their production is not necessarily, nor even usually attributable. And we fearlessly repeat, that were the stimulating agents, which give rise to the sympathetic action, applied to the secreting organs themselves, we are not authorized to believe that the healthy functions of those organs would be in any measure increased. The reverse, we doubt not, would certainly occur.

But why should we dwell any longer on this subject? The principle for which we are contending is a general one. Every vital action, of whatever form or description, we repeat again, is the result of stimulation. And a vast majority of these actions are of necessity the product of remote stimulation. Indeed the effects of this sort of stimulation are seen in all parts of the body; those of proximate or immediate in comparatively but few parts. And this is true, whether the system be in health or disease, as might be easily shown, had we time and room to prepare and exhibit an analysis of the subject. In the course of such analysis we could make it clearly appear, that the parts of the body are few in which it is even possible for proximate stimulation to occur.

Such are the remarks which we have deemed it necessary to premise, that we may be the more easily and clearly understood, in the exhibition we are about to offer of the sympathetic theory of impregnation. And we trust they will prove sufficient for the purpose intended. As further preparatory however to their application, we ask the reader to bear in mind the well known and important fact, to which allusion has been already made, that every organ or part in the generative system of woman sympathizes very strongly with every other part. The commencement and course of the process of impregnation appear to be as follows.

I. The semen masculinum being deposited in the vagina, produces there its specific impression, which can alone be productive of the specific effect intended to be the second step in the process.

II. With this vaginal impression the ovarium next sympathizes, taking on a specific and corresponding mode of action. And that mode is such as, by its secretory and formative character, to give the complete and finishing touch to the ovum—a principal element of which is the "germinal vesicle."

III. The third form of action that shows itself sympathetically, is in the fallopian tubes, which by becoming rigid, and more highly charged with arterial blood, and of course with vital energy, and undergoing other changes in size and figure, pass into a state of preparation to receive the ovum from the ovary, and convey it into the uterus.

IV. Next occur, by sympathy, in the fundus uteri, the changes designed to fit it for the reception, attachment, and general accommodation of the ovum, by the formation of the maternal portion of the membranes, and the placenta. Nor is this all.

On the same principle, and in promotion of the same object, a change occurs in the action of the membrane which lines the neck of the uterus, the design of which is, the obstruction of the canal leading into the vagina, to prevent the escape of the ovum in that direction.

Nor do the changes connected with impregnation end here. Far from it. The entire female system undergoes a striking change. The mammæ enlarge and soften, the nervous system becomes more sensitive and excitable, uncommon tastes and longings, sentiments and propensities often occur, and, toward the close of gestation, strongly-marked and important changes occur in the external organs of generation. These last changes are preparatory to parturition, which, without them could never be effected. But we must proceed no farther in detail on the subject.

The foregoing, though not all, are some of the leading changes, which, in consequence of impregnation, occur in the genital organs, and in other parts of the system of woman. And it is not easy to decide which of them is the most strikingly interesting and important. Yet the act of *immediate impregnation* excepted, they are all acknowledged to be the offspring of sympathy.

In truth to deny this, or even to question it, would be eminently preposterous. It would involve the weak and extraordinary notion, that the semen masculinum by its *actual presence in every altered part of the body, performs the whole work.* That it stimulates the fallopian tubes, increases their size, changes their shape, expands their fimbriæ, and renders them rigid. That, by its own direct and immediate action, it stimulates the fundus uteri into the preparation it makes to receive, and retain, and accommodate the ovum. That, on the same ground, and by its own immediate influence, it induces the mucous lining of the neck of the uterus to act in such a way as to close the mouth of that organ, the more certainly to prevent the loose ovum from escaping into the vagina. And that it extends in substance to the mammæ, and produces in them the changes they undergo.

All these changes we say must be produced by either the actual presence of the semen masculinum *in*, and its action immediately *on* the organs changed, or by *sympathy* with the primitive change produced by it, as most other persons allege, in the ovarium, but, as we believe, in the vagina, where *we know* that it is deposited. That any physiologist, we repeat, can even sportively imagine, much less seriously contend for, the former of these modes of the production of such changes, we believe to be impossible. Some form of the latter therefore must necessarily be adopted, or the subject dismissed, as altogether inscrutable.

If then sympathy produces all the other changes which occur after impregnation, in various parts of the female system —in the fallopian tubes, the fundus uteri, the body and neck of the uterus, the mammæ, the external organs of generation, and elsewhere—if sympathy does all this, may it not also produce (what would seem to be much more easily done) that change, denominated impregnation, which occurs in a small portion of the ovarium? A reply to this question in the affirmative is but a simple inference from the *major* to the *minor* —a conclusion that, if sympathy can accomplish the former achievement, great and complicated as it is, *a fortiori* it can accomplish the latter.

We defy the ablest and most ingenious opponent of the sympathetic doctrine to render even a specious reason why, if for the production of impregnation, the *presence* of semen masculinum on the ovarium be *indispensable*, it is not equally so at the mammæ, and other parts of the female system, to produce in them the changes which follow. Nor will any one, on fair terms, accept the challenge, from a full conviction that the day will go against him. We, on the contrary, hold ourselves prepared to submit to our readers as plausible and sound reasons why semen masculinum should be present in the mammæ, to produce in them the secretion of milk, and the other changes in action and condition which they undergo during pregnancy, as the ablest humoralist can give, why medicinally substances of any description must be *formally* mingled with the blood, in order to prove either beneficial or prejudicial to deranged organs.

At the risk of being deemed unnecessarily wordy, we again express our regret, that a want of room prevents us from republishing Dr. Haighton's account of his experiments in defence of the sympathetic theory of impregnation. As far as we are informed, these experiments have never received from any quarter, a reply of force sufficient to shake them in the slightest degree; and, as already intimated, they are, in our own opinion, conclusive of the controversy.

Were it admissible for us to protract this discussion any farther, we could also draw from the vegetable, and the lowest orders of the animal kingdom, sundry analogies highly favorable to the same theory. But we content ourselves, for the present, with a brief statement of the following curious and interesting fact. And for its authentiaity we refer to writers on entomology.

The aphis (cabbage-fly, or rather cabbage louse) produces in the course of the year several generations of descendants—one of them *oviparous*, and all the others *viviparous.*

The oviparous is hatched in the spring—say in April or May. This is followed suscessiyely by the viviparous, throughout the summer and early autumn, when, at a more advanced period, another generation, or deposition of eggs occurs, which lies dormant during the winter, and is hatched again in the following spring. And thus, from year to year, goes on their reproduction. But the most striking and extraordinary feature of the process remains to be mentioned, and is as follows.

The first spring-brood (that we mean which is of oviparous blrth) consists of males and females. These unite and celebrate their loves, like other insects, and the males immediately die.

The next generation is viviparous, and all are females. This generation, without sexual intercourse (the males being all dead) gives birth to another generation, which, in like manner, consists also entirely of females. This race produces a a third, still viviparous, and still of the same sex. And thus viviparous reproduction proceeds, until some time in the autumn, when another crop of eggs is deposited to be awakened into active life, and moulded into due form, by the returning spring. Such are the facts of the case. Now for the philoso phy.

Here are several successive generations (we think from five to seven) where the female has become pregnant, and given birth to offspring, without copulation. Whence, we ask, is derived the male semen, to come into contact with the ovarium, as an indispensable event, and produce impregnation? To the advocates of the contact-hypothesis the question is addressed, for such solution as may await it, from their *wisdom* and *philosophy.*

We ourselves very frankly confess, that we have neither the one nor the other adapted to the subject. To us the event is therefore inexplicable; yet not more so than every other connected with generation. For the entire process is inexplicable. And in real inexplicability there are no degrees. The whole achievement is a dark mass. We understand no better how impregnation is effected with copulation, than without it. The impregnation of the female aphis after the death of the male, and therefore *without* his immediate influence, we understand just as well, as we do the impregnation of any other female animal *with* the influence of the male, and during his lifetime. Nor is there, as we confidently believe, any more necessity, as regards impregnation, for the semen masculinum to come into contact with the ovarium in woman, nor any more truth in the notion that it does so, than there is in the case of the female aphis, in those impregnations which occur in her, after the death of all the males of her race.

In no other case, except that of the female aphis, do we contend for the existence of *conclusive* evidence that impregnation can be and is effected, without the *possibility* of contact taking place between the male seminal fluid, and the ovarium. But in that case the impossibility is complete.

Analogy therefore, amounting to strong probability, which, under a deficiency of positive evidence, has great weight, forcibly indicates that a similar condition of things may prevail in the human subject—similar, we mean, as to the actual impossibility of contact occurring between the semen masculinum and the ovarium. And our reasons for believing that it positively does prevail, have been already submitted to the consideration ef the reader. A few thoughts on the probable purposes and uses of *menstruation* shall close this article.

Though we believe it to be, with physiologists, the prevailing opinion, that the function of menstruation is peculiar to woman (the females of all the inferior animals being strictly *amenstruous*,) yet it is asserted by naturalists not unworthy of credit, whose opportunities to become acquainted with the matter in question have been favorable, that the case is otherwise; and that the females of the ape tribes are menstruant.

Another attribute deemed peculiar to woman is, that she admits, at all times the sexual embrace, the females of the inferior animals admitting it only periodically, and generally after long periods of intervening abstinence. But to this usage also the female ape is represented as an exception. She is said to receive the male, if not at *all* times, at least at much shorter intervals, than the females of other tribes of the inferior animals. Of these facts (for the truth of which however we do not fully and definitively vouch) the legitimate bearing will presently appear. But previously to this a remark or two on the nature of the menstrual function may not be amiss. And the object at which we immediately aim, at this moment, is sufficiently embraced in a correct reply to the following question.

Whether is menstruation a hemorrhagic, or a secretory function? in other and plainer words, whether is the menstrual fluid real blood, or something prepared from the blood, by a secretory process? To this interrogatory our reply is brief. We deem menstruation a secretory function, for the following reasons.

1. The menstrual fluid is not blood. Why? Because it does not, like blood, coagulate; nor does it, of course, contain any true fibrin, the master element, and the true cause of the coagulation of blood. It consists of only red globules, gelatine, and serum, which, of themselves do not, we repeat, constitute blood—wanting, as they do, the most important element in the composition of that fluid. In cases of real uterine hemorrhagy, the fluid discharged does coagulate, contains fibrin, and is therefore genuine blood. In evidence of its true coagulation, it is discharged in clots. But the menstrual discharge, in a natural and healthy condition, is always fluid.

2. The odor of the menstrual fluid is peculiar—we might say specific—and altogether different from that of blood. It is truly as *sui generis,* as the odor of assafœtida, castor, or urine.

3. The menstrual is a *dead* liquid, blood a *living* one. When chemically analyzed, the former affords results quite different from those of the latter, under similar treatment.

4. Like other glandular bodies, the uterus receives more blood than is requisite for its nourishment. Hence like such bodies it frees itself from the superfluous portion of that fluid, not by a hemorrhage, but a secretory process. All natural and healthy discharges of fluids from the human body are the results of secretion.

Another question respecting menstruation is frequently asked, and differently answered by different physiologists. Whether is it a healthy and natural, or a morbid and unnatural process? Answer. The former certainly. It is as necessary to the health of woman, and therefore itself as healthful a process, as is that of digestion or the secretion of bile. Amenstruous women are never healthy. Is it possible then for deranged action in an important and influential organ to contribute to the sound condition of the whole body? An affirmative reply to this question from the lips or the pen of an enlightened physiologist would greatly surprise us. We ourselves reply, that we deem an issue of the kind physically *impossible;* because it is contrary to all we know of the laws and economy of nature. To contend or even admit, that an unsound *part* can tend to or effectuate the production of a sound *whole,* would amount to as positive an absurdity, as any other self-contradiction that can be uttered or conceived.

True; amenstruous women do occasionally bear children. But that avails not. They rarely if ever bear healthy children; and are *never* healthy during gestation. All observation compels us to believe that menstruation is essential to the perfectly sound and efficient condition of the generative organs and their functions in woman. In fact the notion that the healthy propagation of our race depends on morbid action in the propagating organs is so utterly preposterous, as to be unworthy of a serious effort to refute it. It is so self contradictory as to set nature at war with herself for the purpose of insuring a salutary result—of actuully *bringing physical good out of physical evil.* As reasonably and well might the advocates of this hypothesis contend, that some morbid affection of his genital organs renders man also more competent to the propagation of his race, than he would be without it. Indeed reasons not easily refuted might be given, why, toward the production of a sound and vigorous offspring, the health and good condition of women should contribute even more than those of men. Corroborative, and even confirmatory of this, if we mistake not, is the result of observation in all enlightened nations, where the subject has received the attention it deserves. In Turkey, Persia, and Arabia, the belief in the doctrine here suggested is universal among the more intelligent and observing classes of society. The Arabs apply it in a particular manner to their domestic animals. Hence young and sound Arab mares of the *first order* are rarely if ever sold, or given as presents. Not one has ever been imported into America; and few if any into Europe. Males on the contrary are habitually sold and exported as presents.

As respects the design and usefulness of menstruation, some light may probably be thrown on them by a comparison of the process with an analogous event which occurs in the females of many sorts, perhaps indeed of all sorts of inferior animals of the higher orders. We refer to what is called the *rut* or *heat*, a condition of the genital organs which appears to be designed for two purposes—to awaken and vivify the sexual appetite in the female, so that she may freely admit the male; and to prepare her organs for the work of impregnation. We consider these to be the intention and use of the rut in the females of the inferior animals, because it is during the period of the existence of that state of the parts, and no other, that they admit the male to sexual connexion with them, or are susceptible of impregnation, were they to do so. And, as already mentioned, the rut in the females of inferior animals is strongly analogous to menstruation in woman. So similar indeed are the two conditions to each other, that the mere *discharge* of the menstrual fluid constitutes their only appreciable difference.

The rut therefore, we say, is and does to the females of the inferior animals what menstruation is and does to human females. It enlivens and heightens in them the sexual appetite, and prepares them the better for the process of impregnation. A few details on this topic will demonstrate, if we mistake not, the truth of our position. Some of these details we derive from the effects or accompaniments of the rut in the females of our domestic animals. And they are strongly analogous to the phenomena of menstruation.

During the time of menstruation, it is well known, from ocular inspection, that the ovaries and whole generative system of woman are unusually engorged with arterial blood. So is the generative system of the cow, the mare, the ewe, and all other female animals, during the time of their rut or heat. And, during the same period, though the external organs of our domestic female animals do not discharge a genuine menstrual fluid, in the usual acceptation of the term, they discharge something strongly resembling it. The fluid is neither blood-like, nor large in quantity; but it is very perceptible to the eye, frequently witnessed, and not unfrequently tinged with blood.

Thus analogous are the phenomena of menstruation and rut. And in their effects on the female generative system, as regards its susceptibility of impregnation, they are also similar. As already stated, the females of our domestic animals, as well as of the deer, the rabbit, the hare, and others both wild and tame, are impregnated only, and admit the male only, during their heat, or rutting season. And, though woman admits the sexual embrace at all seasons, she does so with more warmth and strength of feeling just before and after the period of menstruation, than at any other. And she is then, in like manner, most susceptible of impregnation. Hence married women, whose sexual intercourse is uninterrupted, begin habitually their count of pregnancy from about the close of a menstrual period. And their calculation is rarely incorrect.

But wherefore does woman permit and enjoy the sexual embrace, at all times—other female animals doing so periodically, and only at long intervals? The answer is deemed obvious and easy. *Because she frequently menstruates.* On each occasion her genital system, especially the ovaries and uterus, becomes abundantly injected with arterial blood—an event which, whenever or wherever it *healthily* takes place, always refreshes the part, invigorates its vital properties, and fits it for a more energetic and efficient performance of its functions. And the intervals between the menstrual periods are so brief, that the condition of augmented feeling and vigor imparted to the genital organs, at a preceding period, is not all lost, before the occurrence of a subsequent one. Hence the constant sexual feeling of woman, and the permanency of her fitness for impregnation—marked, however, as already stated, by different degrees of these attributes, at different times.

Although we now, for the first time, publish these views on the nature, effects, and uses of menstruation, yet have we entertained and defended them, both publicly and privately, for many years. Nor, as far as our knowledge and recollection serve us, have they been published or entertained by any body else.

We claim them, therefore, as our own, assume the responsibility of any errors they may contain, and deem ourselves entitled to such credit and consideration as they may be thought calculated to confer. Their correctness receives additional confirmation from the two following facts. In woman the sexual appetite greatly declines—is at times entirely extinguished, and she ceases to be fruitful, as soon as her catamenial function disappears. Without any farther illustration or proof, therefore, we submit them to the unprejudiced judgment of the reader, not without some hope, that he will not find them altogether unworthy of his attention, nor peradventure of his approval.

